# Healthy Lifestyle During the Midlife Is Prospectively Associated With Less Subclinical Carotid Atherosclerosis: The Study of Women's Health Across the Nation

**DOI:** 10.1161/JAHA.118.010405

**Published:** 2018-11-28

**Authors:** Dongqing Wang, Elizabeth A. Jackson, Carrie A. Karvonen‐Gutierrez, Michael R. Elliott, Siobán D. Harlow, Michelle M. Hood, Carol A. Derby, Barbara Sternfeld, Imke Janssen, Sybil L. Crawford, Mei‐Hua Huang, Samar R. El Khoudary, Claudia U. Chae, Ana Baylin

**Affiliations:** ^1^ Department of Epidemiology University of Michigan Ann Arbor MI; ^2^ Division of Cardiovascular Disease University of Alabama at Birmingham AL; ^3^ Department of Biostatistics University of Michigan Ann Arbor MI; ^4^ Survey Research Center University of Michigan Ann Arbor MI; ^5^ Department of Neurology Albert Einstein College of Medicine Bronx NY; ^6^ Department of Epidemiology and Population Health Albert Einstein College of Medicine Bronx NY; ^7^ Division of Research Kaiser Permanente Oakland CA; ^8^ Department of Preventive Medicine Rush University Medical Center Chicago IL; ^9^ Medical School University of Massachusetts Worcester MA; ^10^ Division of Geriatrics University of California Los Angeles Los Angeles CA; ^11^ Department of Epidemiology University of Pittsburgh PA; ^12^ Cardiology Division Massachusetts General Hospital Boston MA; ^13^ Department of Nutritional Sciences University of Michigan Ann Arbor MI

**Keywords:** atherosclerosis, cardiovascular disease, lifestyle, risk factors, women, Epidemiology, Cardiovascular Disease, Lifestyle, Risk Factors, Women

## Abstract

**Background:**

Measures of subclinical atherosclerosis are predictors of future cardiovascular outcomes as well as of physical and cognitive functioning. The menopausal transition is associated with accelerated progression of atherosclerosis in women. The prospective association between a healthy lifestyle during the midlife and subclinical atherosclerosis is unclear.

**Methods and Results:**

Self‐reported data on smoking, diet, and physical activity from 1143 women in the Study of Women's Health Across the Nation were used to construct a 10‐year average Healthy Lifestyle Score (HLS) during the midlife. Markers of subclinical atherosclerosis were measured 14 years after baseline and included common carotid artery intima‐media thickness (CCA‐IMT), adventitial diameter (CCA‐AD), and carotid plaque. The associations of average HLS with CCA‐IMT and CCA‐AD were estimated using linear models; the association of average HLS with carotid plaque was estimated using cumulative logit models. Average HLS was associated with smaller CCA‐IMT and CCA‐AD in the fully adjusted models (*P=*0.0031 and <0.001, respectively). Compared with participants in the lowest HLS level, those in the highest level had 0.024 mm smaller CCA‐IMT (95% confidence interval: −0.048, 0.000), which equals 17% of the SD of CCA‐IMT, and 0.16 mm smaller CCA‐AD (95% confidence interval: −0.27, −0.04), which equals 24% of the SD of CCA‐AD. Among the 3 components of the HLS, abstinence from smoking had the strongest association with subclinical atherosclerosis.

**Conclusions:**

Healthy lifestyle during the menopausal transition is associated with less subclinical atherosclerosis, highlighting the growing recognition that the midlife is a critical window for cardiovascular prevention in women.

**Clinical Perspective**

**What Is New?**

- Women who have a healthy lifestyle, composed of abstinence from smoking, healthy diet, and engagement in regular physical activity, during the menopausal transition have lower levels of subclinical atherosclerosis later in their life.
- The prevalence of these healthy behaviors is extremely low in midlife women.
- Among the 3 components of this healthy lifestyle, abstinence from smoking has the strongest association with subclinical atherosclerosis.

**What Are the Clinical Implications?**

- This work highlights the growing recognition that the midlife is a critical window for cardiovascular prevention in women.
- Prevention of future cardiovascular disease among women undergoing the menopausal transition should focus on modifiable health behaviors including smoking, diet, and physical activity.

## Introduction

Cardiovascular disease (CVD) is the leading cause of morbidity and mortality in the United States in women as well as in men.^1^ Women experience a steeper increase in CVD risk during and after the menopausal transition relative to before menopause.^1^, ^2^, ^3^ Menopause is also associated with several adverse changes of cardiovascular risk factors independently of chronological aging, such as increased levels of total cholesterol, low‐density lipoprotein (LDL) cholesterol, and apolipoprotein B.^4^ This suggests that the midlife may be an especially relevant period for cardiovascular risk assessment and prevention in women.

Subclinical atherosclerosis is closely related to the onset of clinically apparent CVD and to CVD mortality. The development of subclinical atherosclerosis typically precedes the occurrence of clinical CVD by years to decades.^5^ Measures of subclinical atherosclerosis of the common carotid artery (CCA), such as intima‐media thickness (IMT), adventitial diameter (AD), and carotid plaque, are clinically important predictors of future CVD events^6^, ^7^, ^8^ and are useful in quantifying CVD risk in asymptomatic individuals without clinically diagnosed CVD.^6^, ^8^, ^9^ In addition to being predictive of clinical outcomes, measures of subclinical carotid atherosclerosis are also associated with poorer physical and cognitive functioning in old age independent of clinical CVD.^10^, ^11^, ^12^ The distribution and determinants of subclinical atherosclerosis differ substantially by age and sex.^13^ It has been shown previously that the menopausal transition is associated with accelerated progression of subclinical carotid atherosclerosis.^14^, ^15^

Abstinence from smoking, adoption of a healthy diet, and engagement in regular physical activity are 3 well‐known modifiable behavioral factors that are considered part of a heart‐healthy lifestyle. Prior studies have found inverse associations between an overall healthy lifestyle and various CVD outcomes, including coronary heart disease,^16^ myocardial infarction,^17^ and CVD‐related mortality.^18^ However, to the best of our knowledge, no study has examined the prospective association between the long‐term lifestyle in midlife women and subclinical atherosclerosis. Because of the association of subclinical atherosclerosis with future clinical CVD and physical/cognitive functioning, as well as the accelerated progression of atherosclerosis during the menopausal transition, the potential effect of modifiable health behaviors on subclinical atherosclerosis in midlife women warrants further investigation as the midlife may be a critical window of opportunity for prevention. In fact, a prior study shows that a lifestyle education program targeting diet and physical activity might be able to slow the menopause‐related progression of atherosclerosis.^14^ Therefore, we aimed to use data from the SWAN (Study of Women's Health Across the Nation) to create a composite healthy lifestyle score (HLS) from 3 behavioral CVD risk factors that are largely modifiable (smoking, diet quality, and physical activity) and to evaluate the prospective association between the HLS during the midlife and measures of subclinical carotid atherosclerosis. We also aimed to explore the independent association between each component of the HLS and subclinical carotid atherosclerosis.

## Methods

The data, analytic methods, and study materials will not be made available to other researchers for purposes of reproducing the results or replicating the procedure.

### Study Design and Study Population

The SWAN is an ongoing, multicenter, multiethnic, prospective cohort study initiated in 1996 to study the natural history of the menopausal transition. Details of the SWAN protocol have been described previously.^19^ Briefly, SWAN participants were recruited from 7 sites across the United States: (1) Boston, Massachusetts; (2) Chicago, Illinois; (3) Southeastern Michigan; (4) Los Angeles, California; (5) Newark, New Jersey; (6) Pittsburgh, Pennsylvania; and (7) Oakland, California. Women who identified themselves as black (Pittsburgh, Chicago, Detroit, and Boston), Chinese (Oakland), Japanese (Los Angeles), Hispanic (Newark), or non‐Hispanic white (all sites) were enrolled. Baseline eligibility criteria included the following: age 42 to 52 years, having an intact uterus and at least 1 ovary, not being pregnant or lactating, not using oral contraceptives or hormone therapy in the past 3 months, and having at least 1 menstrual cycle in the past 3 months. The initial sample size at baseline was 3302. Clinic assessments began in 1996 and the participants have been followed up for 15 approximately annual examinations through the most recent visit in 2015–2016. The SWAN protocols were approved by the Institutional Review Board at each site, and all participants provided written informed consent at each study visit.

**Figure 1:**
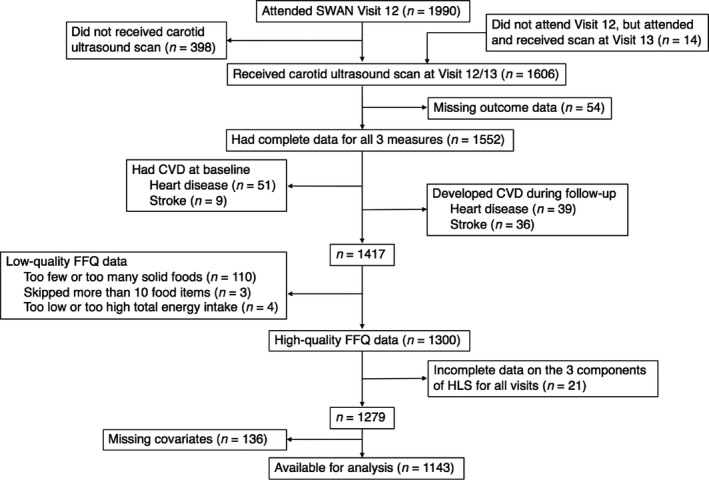
Exclusion criteria of the participants in the Study of Women's Health Across the Nation. CVD indicates cardiovascular disease; FFQ, food frequency questionnaire; HLS, Healthy Lifestyle Score; SWAN, Study of Women's Health Across the Nation.

Carotid ultrasound scans were conducted at 6 sites (except the Los Angeles site) at SWAN follow‐up Visit 12 (2009–2011) or Visit 13 (2011–2013), with the vast majority of scans performed at Visit 12. Among the 2806 women initially enrolled at these 6 sites, 1990 (70.9%) participants attended Visit 12, of whom 1592 (80.0%) had a carotid scan at either Visit 12 or Visit 13. Additionally, 14 women did not attend Visit 12, but attended and received their carotid scan at Visit 13. Thus, a total of 1606 women had a carotid scan at either Visit 12 or Visit 13. From these 1606 participants, we further excluded women who lacked data on the 3 specific measures of carotid atherosclerosis (n=54); who self‐reported having heart disease (n=51) or stroke (n=9) at baseline or who developed heart disease (n=39) or stroke (n=36) during the follow‐up; who reported too few (<4) or too many (>16) numbers of solid foods per day (n=110), skipped more than 10 food items on the food frequency questionnaire (n=3) or reported a total energy intake that was too low (<2092 kJ/day, ie, 500 kcal/d) or too high (>20 920 kJ/d, ie, 5000 kcal/d) (n=4); who had incomplete data on the 3 components of HLS for all visits (n=21); and who had missing data for the major covariates (n=136). After these exclusions, the final analytical sample consisted of 1143 women (Figure 1); 98% (n=1121) of the retained participants received their carotid ultrasound scan at Visit 12.

Comparisons of baseline characteristics of women in the analytic sample (n=1143) with those excluded (n=861) showed that the excluded participants were significantly (*P*<0.05) more likely to be black (42.6% of the excluded women versus 26.2% of the retained women were black) and early perimenopausal (48.7% versus 43.7%), and were significantly more likely to report difficulty paying for basic necessities (48.0% versus 31.0%) and depressive symptoms (28.2% versus 21.8%), to have a higher body mass index (mean: 29.9 kg/m^2^ versus 27.9 kg/m^2^), high blood pressure (37.0% versus 26.2%), impaired fasting glucose (30.3% versus 20.6%), and low HDL cholesterol (41.0% versus 32.6%). At baseline, the excluded participants were significantly less likely to have a college degree (39.5% of the excluded women versus 48.9% of the retained women had a college degree), to be married or living as if married (58.3% versus 70.3%), and to self‐report having excellent/very good overall health (51.3% versus 64.5%). Age at baseline, age at the carotid scan, hormone therapy use during the follow‐up, and baseline proportions of high serum triglycerides, high total cholesterol, and high LDL cholesterol did not differ significantly between the retained and the excluded participants.

### Assessment of Exposures

In a prior study in SWAN, Sternfeld et al created a HLS using smoking, physical activity, and diet quality (quantified by a healthy diet score).^20^ Building on this prior work, we modified the HLS by using the well‐established Alternate Healthy Eating Index (AHEI) to quantify diet quality, while keeping the scoring methods of smoking and physical activity the same as in the original HLS. The AHEI is an a priori dietary index that quantifies the adherence of one's diet to certain dietary guidelines. The AHEI has repeatedly been shown to be predictive of the risk of chronic disease and can better account for the types of fiber, protein, and fats.^21^, ^22^, ^23^

Dietary data were collected at baseline (1996–1997), Visit 5 (2001–2003), and Visit 9 (2005–2007) using a modification of the 1995 version of the Block food frequency questionnaire (FFQ), which has previously been validated against dietary records^24^ and 24‐hour recalls.^25^ Briefly, among women, the deattenuated correlation coefficients (ie, after removing the random, within‐person errors)^26^ between the Block FFQ and 24‐hour recalls for total energy intake, protein, carbohydrate, total fat, saturated fat, monounsaturated fat, and polyunsaturated fat were 0.45, 0.53, 0.66, 0.67, 0.65, 0.60, and 0.48, respectively.^25^ The FFQ used in SWAN included 103 food items, including 83 solid food items and 20 beverage food items. The FFQ was administered by trained personnel. The participants were asked how often, on average, they consumed each food of a standard portion size during the past year. Up to 9 possible responses were available for each food item: <once/mo or never, once/mo, 2 to 3 times/mo, once/wk, twice/wk, 3 to 4 times/wk, 5 to 6 times/wk, once/d, and ≥twice/d. We further transformed the responses into semicontinuous variables representing servings per day, with values 0.016, 0.03, 0.08, 0.14, 0.28, 0.5, 0.79, 1, and 2 for the 9 responses, respectively. Intake of energy and nutrients was computed by multiplying the consumption frequency of each food by the corresponding nutrient content.

For each visit, we calculated the AHEI for each participant. The AHEI includes 9 components of foods and nutrients intake (Table S1). The scores of the 9 components were summed to obtain the total AHEI score, which ranged from 2.5 (worst diet) to 87.5 (best diet). We further collapsed each participant's AHEI score and gave a score of 2 when the participant's AHEI score was in the top tertile of the study population, a score of 1 when the AHEI score was in the middle tertile, and a score of 0 when the AHEI score was in the bottom tertile.

Current guidelines recommend that adults should pursue at least 150 min/wk of moderate‐intensity physical activity for substantial health benefits.^27^ Physical activity was evaluated from the sports and exercise questions on the validated Kaiser Physical Activity Survey^28^ to determine whether this recommendation was met.^20^ We gave a score of 2 (fully meeting the recommendation) to those who played sports or exercised more than once a week, for at least 2 h/wk for at least 9 months during the past year and with at least a moderate increase in heart rate and breathing. We gave a score of 1 (partially meeting the recommendation) to those who played sports or exercised more than once a month but no more than once a week, or to those who played sports or exercised more than once a week but did not satisfy other criteria to qualify for a score of 2. We gave a score of 0 (not meeting the recommendation) to those who played sports or exercised no more than once a month. Data of physical activity at baseline, Visit 5, and Visit 9 were used (ie, the same time points as the available dietary data).

Standardized questions from the American Thoracic Association^29^ were used to collect information on smoking status. We gave a score of 2 to never smoking, a score of 1 to former smoking, and a score of 0 to current smoking. Data at baseline, Visit 5, and Visit 9 were used.

#### Healthy lifestyle score

To calculate the HLS, we computed the arithmetic sum (ie, without weighting) of the scores for the individual components of smoking, physical activity, and diet quality to create visit‐specific HLS, with a possible range of 0 to 6 for each visit (baseline, Visit 5, and Visit 9). The visit‐specific scores were then averaged across all nonmissing visits to create the average HLS. To uncover potential nonlinear associations, we further divided the average HLS into 4 levels: 0 to 2, >2 to 3, >3 to 4, and >4 to 6, which approximated the quartiles of the HLS distribution.

### Assessment of Outcomes

The details of the carotid ultrasound measurements have been described elsewhere.^30^, ^31^ Briefly, at all SWAN sites except the Los Angeles site, centrally trained and certified sonographers obtained carotid ultrasound images at Visit 12 (and Visit 13 for a small group of participants), using a Terason t3000 Ultrasound System (Teratech Corp, Burlington, MA) equipped with a variable frequency (5–12 MHz) linear array transducer. Two digitized images were obtained of each of the left and right distal CCA. From each of these 4 images, using the AMS semiautomated edge detection software,^32^ near and far wall IMT measures of the CCA were obtained by electronically tracing the lumen–intima interface and the media–adventitia interface across a 1‐cm segment proximal to the carotid bulb; 1 measurement was generated for each pixel over the area, for a total of ≈140 measures for each segment. The average and maximal values for these measures were recorded for all 4 images, with the mean of the maximal readings of all 4 images used in the analyses. AD of the CCA was measured as the distance from the adventitial–medial interface on the near wall to the medial–adventitial interface on the far wall at end‐diastole across the same CCA segments used for IMT measurement. The mean value of the average readings was used in the analyses. Carotid scan images were read centrally at the SWAN Ultrasound Reading Center (University of Pittsburgh Ultrasound Research Laboratory). Sonographers at each site evaluated the presence and extent of plaque in each of 5 segments of the left and right carotid artery (distal and proximal CCA, carotid bulb, and proximal internal and external carotid arteries). A plaque was defined as a distinct area protruding into the vessel lumen that was at least 50% thicker than the adjacent IMT. For each segment, the degree of the plaque was graded between 0 (no observable plaque) to 3 (plaque obstructing ≥50% of the luminal diameter of the vessel). The grades from all segments of the combined left and right carotid artery were summed to create the plaque index.^33^ Technicians at the 6 study sites were trained by the University of Pittsburgh Ultrasound Research Laboratory and monitored during the study period for reliability. Reproducibility of IMT measures was good to excellent with an intraclass correlation coefficient between sonographers of ≥0.77, and between readers of >0.90. The plaque index was similarly reliable with an intraclass correlation ranging from 0.86 to 0.93.^34^

The 3 outcomes of this study were the intima‐media thickness of the common carotid artery (CCA‐IMT), the adventitial diameter of the common carotid artery (CCA‐AD), and the extent of carotid plaque (categorized by carotid plaque index). We treated CCA‐IMT and CCA‐AD as continuous outcome variables. We collapsed carotid plaque index into none (0), moderate (1), and high (≥2) and treated it as a categorical (ordinal) outcome variable.

### Assessment of Covariates

Self‐reported covariates at baseline included age (continuous), race/ethnicity (black, Hispanic, Chinese, or non‐Hispanic white), education level (≤ high school, some college, or college degree/postcollege), financial strain (somewhat/very hard paying for basics, or not hard paying for basics), marital status (single/never married, married/living as if married, or separated/widowed/divorced), self‐rated overall health (excellent/very good, good, or fair/poor), depressive symptoms (dichotomized by the Center for Epidemiological Studies Depression Scale: ≥16 or <16),^35^ and menopausal status based on self‐reported menstrual bleeding patterns (dichotomized as premenopausal or early perimenopausal).^36^ Self‐reported use of hormone therapy during the follow‐up was dichotomized as ever use or never use, with ever use defined as use of hormone therapy at any visit from baseline to the visit of the carotid scan. The presence of hot flash was self‐reported at Visit 12. Weight and height were measured by trained interviewers using a calibrated balance beam scale and a stadiometer, respecitvely, and body mass index (BMI) was calculated as weight in kilograms divided by squared height in meters. Blood pressure was calculated as the average of 2 seated measurements using a standard mercury sphygmomanometer, and blood samples were taken to measure fasting glucose, total cholesterol, serum triglycerides, LDL cholesterol, and HDL cholesterol. Based on harmonized guidelines,^37^ high blood pressure was defined as systolic blood pressure ≥130 mm Hg, or diastolic blood pressure ≥85 mm Hg, or use of at least 1 antihypertensive medication. Impaired fasting glucose was defined as fasting glucose ≥100 mg/dL or use of at least 1 antidiabetic medication. High serum triglycerides was defined as fasting serum triglycerides ≥150 mg/dL. Low serum HDL cholesterol was defined as serum HDL cholesterol <50 mg/dL. Additionally, we considered total cholesterol ≥200 mg/dL as high total cholesterol, and LDL cholesterol ≥130 mg/dL as high LDL cholesterol.

### Statistical Analysis

In the descriptive analysis, we computed the means and SDs for continuous covariates and percentages for categorical covariates for the entire study population as well as stratified by categories of average HLS, adjusting for baseline age. In the multivariate regression analysis, we estimated the association of the levels of the average HLS (0–2; >2–3; >3–4; and >4–6) with CCA‐IMT and CCA‐AD using linear models, and with carotid plaque (high versus moderate versus none) using cumulative logit models (ie, ordinal logistic regression models). No transformations were performed on CCA‐IMT or CCA‐AD. Normality and homoscedasticity of the residuals were checked and satisfied in all linear models. No significant deviation from the proportional odds assumption was observed (*P* values for the score tests >0.05) in the cumulative logit models.^38^

The selection of confounders was based on a priori knowledge of risk factors for subclinical atherosclerosis gained from the literature and supplemented by the empirical exposure‐covariate associations in the study population. We adjusted for baseline covariates, age at the carotid scan, use of hormone therapy from baseline to the visit of the carotid scan, self‐reported hot flash at Visit 12, and the number of missing visits for the HLS. The baseline covariates included race/ethnicity, education level, financial strain, marital status, self‐rated overall health, depressive symptoms, total energy intake, and menopausal status. Physiological risk factors, including BMI, high blood pressure, impaired fasting glucose, serum triglycerides, total cholesterol, HDL cholesterol, LDL cholesterol, use of antilipidemic medications, and use of antihypertensive medications might be both confounders and potential mediators. Therefore, the values of these covariates were additionally adjusted for in separate models. *P* values were computed by using the HLS as a continuous variable in the models. We further explored the association of each component of the HLS (ie, smoking, diet, and physical activity) with subclinical carotid atherosclerosis individually by adjusting for the other 2 components in the same model.

We additionally conducted a series of sensitivity analyses to ensure the robustness of the results. First, we generated a weighted HLS by using the percentage of the coefficient of each individual component to the sum of the coefficients in the models with all components included; to capture the potential J‐shaped association between physical activity and atherosclerosis, we included both the linear and the quadratic terms of physical activity in the creation of the weighed HLS. Second, to rule out selection bias because of the attrition and missing data, we used inverse probability weighting to develop a nonresponse weight for each retained participant based on the baseline predictors of attrition, including race/ethnicity, education level, financial strain, marital status, self‐rated overall health, depressive symptoms, BMI, menopausal status, high blood pressure, impaired fasting glucose, and low HDL cholesterol. We repeated the analyses using the weights in the analysis. Third, we repeated the analyses by adjusting for financial strain, marital status, self‐rated overall health, and depressive symptoms at the visit of the carotid scan rather than at baseline. Fourth, we repeated the analysis by including only the women with HLS data from all 3 time points (baseline, Visit 5, and Visit 9). Fifth, we treated carotid plaque as a binary outcome (carotid plaque index ≥2 versus <2) instead of an ordinal outcome and used logistic regression models. Sixth, we repeated the analysis using the 3 visit‐specific HLSs instead of the average HLS. All analyses were conducted using SAS (Version 9.4, SAS Institute Inc, Cary, NC) at a 2‐sided α level of 0.05. The first author had full access to all the primary data in the study and takes responsibility for the data analysis.

## Results

### Descriptive Analyses

Among the 1143 participants in the analytical sample, those with a high average HLS were more likely to have a college degree, self‐report their overall health to be excellent or very good, have a lower BMI at baseline, be premenopausal at baseline, and use hormone therapy during the follow‐up. Participants with a high average HLS were less likely to be Hispanic or black, experience difficulty paying for basics, be separated/widowed/divorced, experience depressive symptoms, and less likely to have high blood pressure, impaired fasting glucose, high serum triglycerides, high total cholesterol, low HDL cholesterol, and high LDL cholesterol at baseline (Table).

**Table 1:** Characteristics of the Study Population by Category of Average HLS Among the 1143 SWAN Participants

	Total (n=1143)	Categories of Average HLS				*P* Trend^a^
		0 to 2 (n=256)	>2 to 3 (n=293)	>3 to 4 (n=306)	>4 to 6 (n=288)	
Major covariates						
Age at baseline, y^b^	46.31 (2.66)	46.19 (2.59)	46.16 (2.60)	46.21 (2.66)	46.68 (2.75)	0.028
Age at the carotid scan, y^b^	60.15 (2.70)	60.02 (2.65)	59.98 (2.65)	60.06 (2.70)	60.51 (2.77)	0.028
Race and ethnicity						<0.001
Black, %	26.16	34.19	24.25	22.01	18.59	
Hispanic, %	5.77	8.10	8.55	5.23	0.61	
Chinese, %	13.56	1.08	12.70	16.90	24.91	
Non‐Hispanic white, %	54.51	56.63	54.50	55.86	55.89	
Education level						<0.001
High school or less, %	20.65	32.95	21.55	17.24	11.97	
Some college, %	30.45	38.34	35.52	29.91	19.71	
College degree/postcollege, %	48.91	28.70	42.93	52.85	68.32	
Somewhat/very hard to pay for basics, %	30.97	41.46	38.52	29.66	16.52	<0.001
Marital status						<0.001
Single/never married, %	12.95	15.14	10.38	12.33	13.32	
Married/living as if married, %	70.34	62.15	74.93	73.23	73.61	
Separated/widowed/divorced, %	16.71	22.71	14.69	14.44	13.06	
Self‐rated overall health						<0.001
Excellent/very good, %	64.48	52.86	60.20	65.45	77.99	
Good, %	26.60	37.76	30.52	25.12	16.67	
Fair/poor, %	8.92	9.38	9.28	9.43	5.34	
CES‐D scale ≥16, %	21.78	32.29	24.57	22.08	12.47	<0.001
Total energy intake, kJ/d	7606.14 (2736.29)	7919.01 (2820.98)	7439.07 (2772.32)	7546.89 (2477.81)	7641.78 (2588.72)	0.79
BMI, kg/m²	27.91 (6.83)	29.52 (7.16)	28.83 (6.77)	27.65 (6.41)	25.09 (4.73)	<0.001
Smoking status						
Never, %	62.84	21.26	66.55	73.86	84.03	<0.001
Past, %	25.42	37.40	27.30	22.55	15.97	
Current, %	11.74	41.34	6.14	3.59	0.00	
Menopausal status						0.0074
Early perimenopausal, %	43.66	50.24	44.78	38.46	35.97	
Premenopausal, %	56.34	49.76	55.22	61.54	64.03	
Hormone therapy use (ever), %^c^	42.78	39.59	41.45	40.07	43.44	0.047
Self‐reported hot flash, %^d^	81.36	83.59	79.52	80.07	82.64	0.85
Number of missing visits						<0.001
0, %	58.97	44.99	58.86	62.84	75.58	
1, %	26.16	33.39	27.51	24.49	16.73	
2, %	14.87	21.62	13.63	12.68	7.68	
High blood pressure, %	26.16	28.89	23.13	28.02	19.55	0.0084
Use of antihypertensive medications, %	10.85	11.33	11.95	10.46	9.72	0.47
Impaired fasting glucose, %	20.56	24.12	23.06	18.99	11.54	<0.001
Serum triglycerides, mg/dL	104.85 (56.54)	116.38 (60.01)	109.20 (59.64)	98.19 (53.67)	97.25 (50.97)	<0.001
High serum triglycerides, %	16.89	23.45	17.20	16.63	10.61	<0.001
Total cholesterol, mg/dL	192.72 (33.24)	195.64 (34.56)	197.60 (33.76)	190.57 (32.09)	187.45 (31.90)	<0.001
High total cholesterol, %	38.85	41.80	44.37	36.60	32.99	0.013
HDL cholesterol, mg/dL	56.57 (13.67)	52.13 (13.02)	56.20 (13.91)	57.98 (13.10)	59.39 (13.63)	<0.001
Low HDL cholesterol, %	32.63	46.09	33.45	28.43	24.31	<0.001
LDL cholesterol, mg/dL	115.17 (29.84)	120.23 (31.26)	119.54 (30.44)	112.95 (28.22)	108.60 (28.18)	<0.001
High LDL cholesterol, %	29.05	34.77	35.49	26.14	20.49	<0.001
Non‐HDL cholesterol, mg/dL	136.15	143.51	141.39	132.59	128.06	<0.001
Use of antilipidemic medications, %	0.26	0.78	0.34	0.00	0.00	0.11
Subclinical atherosclerosis						
CCA‐IMT, mm^d^	0.92 (0.14)	0.96 (0.15)	0.92 (0.12)	0.90 (0.13)	0.88 (0.12)	<0.001
CCA‐AD, mm^d^	7.19 (0.66)	7.34 (0.66)	7.16 (0.62)	7.10 (0.60)	7.02 (0.58)	<0.001
Carotid plaque^d^^,^ e						0.038
None, %	57.39	49.50	55.94	60.10	58.62	
Moderate, %	18.29	17.47	15.34	18.08	22.67	
High, %	24.32	33.04	28.72	21.82	18.71	

Values are means (SDs) for continuous variables and percentages for categorical variables. Values stratified by categories of average HLS are standardized to the baseline age distribution of the study population. Values of polytomous variables may not sum to 100% because of rounding. The variables are the baseline measures unless specified otherwise. AD indicates adventitial diameter; BMI, body mass index; CCA, common carotid artery; CES‐D scale, Center for Epidemiological Studies Depression scale; HDL, high‐density lipoprotein; HLS, Healthy Lifestyle Score; IMT, intima‐media thickness; LDL, low‐density lipoprotein; SWAN, Study of Women's Health Across the Nation.

a Computed by linear models for continuous covariates and logistic models for binary/categorical covariates. The median HLS of a level was assigned to participants in the corresponding level and treated as a continuous variable.

b Values are not age‐standardized.

c Ever use was defined as reported use at any visit from baseline to the visit of the carotid scan.

d Measured either at Visit 12 (2009–2011) or Visit 13 (2011–2013).

e None: carotid plaque index=0; moderate: carotid plaque index=1; high: carotid plaque index ≥2.

The HLS scores were relatively stable over time. The Spearman correlation coefficient for the HLS score was 0.66 between baseline and Visit 5, 0.70 between Visit 5 and Visit 9, and 0.66 between baseline and Visit 9. Smoking status did not appear to change considerably over time, as 93.4% of the study population did not report a change in smoking status (in terms of current, past, and never) from baseline to Visit 9. Over half (54.2%) of the study population reported a change in their physical activity status (in terms of fully meeting, partially meeting, and not meeting the recommendation) from baseline to Visit 9. The Alternate Healthy Eating Index (AHEI) scores were moderately stable over time. The Pearson correlation coefficient of the AHEI score was 0.63 between baseline and Visit 5, 0.69 between Visit 5 and Visit 9, and 0.62 between baseline and Visit 9. While 711 participants (62.2% of the study population) remained as never smokers at all 3 visits, only 204 (17.8% of the study population) consistently stayed in the top tertile of the AHEI scores, and only 82 (7.2% of the study population) self‐reported physical activity status that consistently met the recommendation. Only 19 participants (1.7% of the study population) remained in the top category for all 3 components at all 3 visits.

### HLS and Subclinical Carotid Atherosclerosis

**Figure 2:**
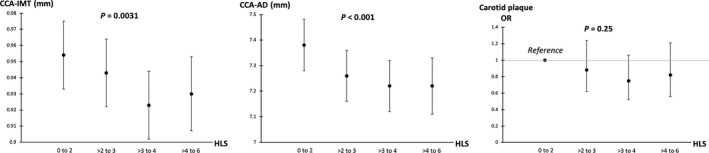
Average HLS and measures of subclinical carotid atherosclerosis among 1143 SWAN participants. Values are least squares means (95% CIs) for CCA‐IMT/CCA‐AD from linear models and odds ratios (95% CIs) for carotid plaque (high vs moderate vs none) from cumulative logit models. *P* values were computed by using the HLS as a continuous variable. Models were adjusted for age at the carotid scan (continuous), race/ethnicity (black, Hispanic, Chinese, or non‐Hispanic white), education (≤ high school, some college, or college degree/postcollege), financial strain (somewhat/very hard paying for basics, or not hard paying for basics), marital status (single/never married, married/living as if married, or separated/widowed/divorced), self‐rated overall health (excellent/very good, good, or fair/poor), Center for Epidemiological Studies Depression scale (≥16 or <16), total energy intake (continuous), menopausal status (premenopausal or early perimenopausal), use of hormone therapy during the follow‐up (ever or never), hot flash at Visit 12 (binary), number of missing visits for HLS (0, 1, or 2), body mass index (continuous), high blood pressure (binary), impaired fasting glucose (binary), serum triglycerides (continuous), total cholesterol (continuous), HDL cholesterol (continuous), LDL cholesterol (continuous), use of antilipidemic medications (binary; Visit 12), and use of antihypertensive medications (binary; Visit 12). The baseline covariates were used unless otherwise specified. AD indicates adventitial diameter; CCA, common carotid artery; CI; confidence interval; HDL, high‐density lipoprotein; HLS, Healthy Lifestyle Score; IMT, intima‐media thickness; LDL, low‐density lipoprotein; OR, odds ratio; SWAN, Study of Women's Health Across the Nation.

The average HLS over 10 years of follow‐up was inversely and statistically significantly associated with CCA‐IMT and CCA‐AD (Figure 2 and Table S2). The inverse associations persisted even after adjusting extensively for confounders and physiological risk factors (*P=*0.0031 and <0.001 for CCA‐IMT and CCA‐AD, respectively). In the fully adjusted models, compared with participants in the lowest level of average HLS (ie, 0–2), those in the highest level (ie, >4–6) had a 0.024‐mm smaller CCA‐IMT (95% CI of β coefficient: −0.048, 0.000), a difference that equals 17% of the SD of CCA‐IMT in the analytical sample; they also had a 0.16 mm smaller CCA‐AD (95% CI of β coefficient: −0.27, −0.04), a difference that equals 24% of the SD of CCA‐AD. We also observed an inverse association between average HLS and carotid plaque after adjusting for major confounders (*P*=0.024), although the association failed to retain its statistical significance after additionally adjusting for physiological risk factors (*P*=0.25).

### Components of HLS and Subclinical Carotid Atherosclerosis

We estimated the independent associations of each component of the HLS by adjusting for the other 2 components in the same model (Tables S3 through S5, for smoking, diet quality, and physical activity, respectively). Abstinence from smoking was strongly and inversely associated with all 3 measures of subclinical carotid atherosclerosis (*P*‐trend<0.01 for all 3 outcome measures). Compared with the participants who smoked at some point during the follow‐up, those who remained never smokers had a 0.047 mm smaller CCA‐IMT (95% CI of β coefficient: −0.070, −0.024; a 34% SD difference), a 0.24 mm smaller CCA‐AD (95% CI of β coefficient: −0.35, −0.13; a 36% SD difference), and 49% lower odds of having a higher carotid plaque index (odds ratio=0.51; 95% CI: 0.35, 0.73) (Table S3). We observed an inverse association between average AHEI score and CCA‐AD after adjusting for major confounders and physiological risk factors (except BMI) (*P*‐trend=0.016). The association lost significance after further adjusting for BMI (*P*‐trend=0.11). We observed a marginally significant inverse association between average AHEI score and CCA‐IMT (*P*‐trend=0.067), which also lost significance after further adjusting for baseline BMI (*P*‐trend=0.18). We found no independent association between average AHEI score and carotid plaque (Table S4). Long‐term physical activity status was marginally significantly associated with CCA‐IMT after adjusting for major confounders (*P*‐trend=0.059). The association lost its significance after additionally adjusting for the physiological risk factors. We found no independent association between long‐term physical activity status and CCA‐AD or carotid plaque (Table S5).

### Sensitivity Analyses

Results from the sensitivity analyses were consistent with the primary analyses. The estimates were similar after using the weighted HLS and including a quadratic term for physical activity (Table S6). The results did not change appreciably after accounting for missing data using the nonresponse weights, adjusting for certain covariates at the visit of the carotid scan (instead of at baseline), and treating carotid plaque as a binary outcome. The sensitivity analysis that focused on women with HLS data from all 3 time points showed stronger associations between average HLS and subclinical carotid atherosclerosis compared with the primary analysis. The results were qualitatively similar when using the 3 visit‐specific HLSs as the exposures. The HLSs at baseline and Visit 5 displayed weaker associations with the outcomes compared with the average HLS, whereas the estimates for the HLS at Visit 9 were similar to those for the average HLS (data not shown).

## Discussion

This study evaluated the prospective associations between a composite HLS and measures of subclinical carotid atherosclerosis in midlife women. The HLS includes 3 health behaviors—smoking, diet quality, and physical activity—which are largely modifiable. We found that the level of average HLS during the midlife over 10 years of follow‐up was significantly associated with smaller CCA‐IMT and CCA‐AD, ≈14 years after baseline. Among the 3 individual components of the HLS, abstinence from smoking displayed the strongest inverse associations with subclinical carotid atherosclerosis.

Accumulating evidence from prior studies indicates that measures of subclinical carotid atherosclerosis are important predictors of future CVD events^6^, ^7^, ^8^ and useful indicators of cardiovascular risk in apparently healthy individuals.^6^, ^8^, ^9^ In a meta‐analysis,^7^ Lorenz et al found that, after adjusting for age and sex, every 1‐SD increase in CCA‐IMT was associated with a 26% higher risk of myocardial infarction and a 32% higher risk of stroke. Similarly, using data from the ARIC (Atherosclerosis Risk in Communities) Study, Eigenbrodt et al reported that every 1‐SD increase in CCA‐AD was associated with 18% higher hazard of incident cardiac events and 73% higher odds of prevalent myocardial infarction in women even after adjusting for CCA‐IMT and other CVD risk factors.^39^ Likewise, the presence of carotid plaque is associated with 83%, 210%, and 81% to 196% higher hazard of myocardial infarction,^40^ stroke,^41^ and death from coronary heart disease,^42^, ^43^ respectively, in women without known cardiovascular disease. Furthermore, the extent of subclinical carotid atherosclerosis is associated with poorer physical^10^ and cognitive^10^, ^11^, ^12^ functioning even among individuals without clinical CVD or after adjusting for the intervening clinical CVD outcomes in the analysis.

Smoking, unhealthy diet, and lack of physical activity are 3 well‐known modifiable behavioral risk factors for CVD. In one of the earliest investigations into overall lifestyle factors and CVD, Stampfer et al followed midlife women in the NHS (Nurses’ Health Study) for 14 years for the occurrence of major coronary events.^16^ They found that women who did not smoke, who engaged in moderate‐to‐vigorous physical activity for ≥30 min/d, and who had a healthy diet (scored in the upper 2 quintiles for a diet high in cereal fiber, marine n‐3 fatty acids, folate, the ratio of polyunsaturated‐to‐saturated fatty acids, and low in *trans* fat and glycemic load) had 57% lower odds of a coronary event compared with all the other women who did not have this healthy lifestyle.^16^ Qualitatively similar results indicating an inverse association between a healthy lifestyle and clinical CVD have been reported in other studies.^17^, ^18^ Notably, a recent analysis used data from the NHS and reported that women with 5 low‐risk lifestyle factors (never smoking, normal BMI, moderate‐to‐vigorous physical activity, moderate alcohol intake, and high diet quality) had 74% lower hazard of all‐cause mortality and 82% lower hazard of CVD mortality, compared with those with zero low‐risk factors.^44^ The projected life expectancy at age 50 years comparing women with 5 low‐risk factors with women with zero low‐risk factors was 14 years (95% CI: 11.8, 16.2) longer, 30.8% of which was attributable to reduced CVD death.^44^

The potential effect of a healthy lifestyle on the extent of subclinical atherosclerosis has been evaluated in very few prior studies.^45^, ^46^, ^47^ Moreover, to the best of our knowledge, no study has examined the association between a composite HLS and subclinical carotid atherosclerosis in midlife women undergoing the menopausal transition. There are significant age and sex differences in the distribution and the determinants of subclinical atherosclerosis.^13^ Independent of chronological aging, the menopausal transition represents a vulnerable window of increased cardiovascular risk^1^, ^2^, ^3^ as well as an accelerated progression of subclinical carotid atherosclerosis.^14^, ^15^ As reported in a previous SWAN study, the progression rate of both CCA‐IMT and CCA‐AD increased considerably from the premenopausal stage to the perimenopausal and postmenopausal stage.^15^ In a randomized trial, Wildman et al implemented a 20‐week lifestyle education program to women undergoing the menopausal transition. The program consisted of 15 group meetings led by nutritional and behavioral interventionalists and was aimed at reducing total and saturated fat intake, preventing weight gain, and increasing physical activity. Women in the intervention group had significantly lower body weights and significantly higher daily kilocalories of exercise after receiving the program compared with the control group. The intervention group also had a slower average progression of carotid IMT compared with the control group.^14^ Thus, the midlife represents a critical window for CVD prevention, and women at this life stage may be particularly responsive to the beneficial effect of lifestyle interventions.

We specifically focused on the 3 health behaviors that are largely modifiable while treating other physiological risk factors affected by lifestyle (ie, BMI, blood pressure, blood lipids, and blood glucose) as covariates. Our study is thus different from previous work using the American Heart Association's Life's Simple 7 goals, which also include the management of BMI, blood pressure, blood glucose, and total cholesterol.^5^ Even after adjusting for the lifestyle‐related physiological risk factors, the adherence to a healthy lifestyle composed of abstinence from smoking, healthy diet, and regular engagement in physical activity is inversely associated with atherosclerosis in midlife women. Adherence to a healthy lifestyle was low in this study, as only 1.7% of the study population stayed in the top category for all 3 components at all 3 time points. This observation is consistent with the NHS, which reported that only 3% of the cohort had a low‐risk lifestyle.^16^ The low prevalence of a healthy lifestyle in midlife women highlights the potential for lifestyle interventions aimed at this vulnerable population.

Abstinence from smoking showed the strongest inverse associations with all 3 measures of subclinical carotid atherosclerosis. Cigarette smoking is a major behavioral risk factor associated with atherosclerosis, through mechanisms including thrombosis, dyslipidemia, insulin resistance, vascular inflammation, abnormal vascular growth and angiogenesis, and loss of endothelial homeostatic and regenerative functions.^48^, ^49^ Multiple studies have shown that prolonged smoking confers a higher CVD risk in women than in men.^50^, ^51^, ^52^, ^53^ Existing evidence is unclear regarding the associations of diet and physical activity with subclinical atherosclerosis. We found that diet quality may be associated with smaller CCA‐AD and CCA‐IMT, and that engagement in regular physical activity may be associated with smaller CCA‐IMT. The observed associations for diet and physical activity lost statistical significance after the adjustment for other physiological risk factors, especially BMI. We thus speculate that BMI may partially mediate the potential beneficial effects of healthy diet and regular physical activity. We did not include BMI (or obesity/overweight status) as a separate component of the HLS but instead treated it as a covariate, because it is not a lifestyle behavior per se, but an intermediate health outcome affected by various other lifestyle factors. Formal causal mediation analysis is needed to quantitatively distinguish the direct effects of diet and physical activity and their indirect effects mediated by other physiological risk factors such as BMI. Because of the several exposures, outcomes, and models examined, this study was not completely free from multiple comparisons. We did not quantitatively correct the *P* values for multiple comparisons because all the models were based strictly on a priori hypotheses and the findings were interpreted with caution.

The current study has some important strengths. The use of repeated measures of behavioral risk factors allowed for a more accurate measurement of long‐term lifestyle. Prior studies have shown that using cumulative exposures from repeated measurements may yield stronger associations with disease outcomes than using only the baseline exposure or the most recent exposure.^54^, ^55^ In this study, the average HLS showed stronger associations with the outcomes compared with most of the visit‐specific scores, though the findings were largely similar because of the relative stability of the HLS. The racial/ethnic composition of the study population was more diverse than most prior studies, because SWAN included not only non‐Hispanic white women but also black, Chinese, and Hispanic women, 3 groups that are underrepresented in the literature. We did not have sufficient statistical power to examine effect modification by race/ethnicity, so future multiethnic studies with larger sample sizes are needed to assess whether the association between lifestyle factors and subclinical atherosclerosis differs by racial and ethnic backgrounds.

This study also has some potential limitations. First and foremost, markers of subclinical carotid atherosclerosis were measured only once, at Visit 12/13. Without the baseline measures, we were unable to evaluate the change of subclinical atherosclerosis from baseline to Visit 12/13. Although we restricted the analyses to apparently healthy participants by excluding women with CVD at baseline or developed CVD during the follow‐up, it is still possible that some women may already have substantial degrees of carotid atherosclerosis before baseline. Therefore, we could not pinpoint the midlife to be the most causally relevant period for the effect of lifestyle. Second, although the prospective nature of the exposures and the asymptomatic nature of the outcomes reduced the extent of reverse causation, it could not be completely ruled out. It was possible that some participants with subclinical atherosclerosis also subsequently had other more detectable health conditions such as abnormal blood lipids or high blood pressure and would thus intentionally improve their lifestyle; this may partially explain the J‐shaped association observed for the highest HLS level for which the outcome measures were similar or even higher than those observed for the second highest HLS level. Another possible reason for the J‐shaped association is that some physiological covariates adjusted for in the full model, such as BMI, blood pressure, blood glucose, and blood lipids, may partially mediate the effect for the highest HLS level, because the J‐shaped tendency was not evident in the crude or partially adjusted models. Third, the exposure data inevitably had some measurement error, especially for the self‐reported data of physical activity and dietary intake. However, both the physical activity questionnaire^28^ and the FFQ^24^, ^25^ used in SWAN have been validated in similar populations. Also, the use of up to 3 repeated measures of exposures considerably reduced the measurement error. Fourth, because the lifestyle data were not available after Visit 9, we were not able to capture the lifestyle exposures between Visit 9 and Visit 12/13. However, 74% of the participants were postmenopausal by Visit 9, so the average HLS from baseline to Visit 9 still reasonably represents the lifestyle during the menopausal transition.

In conclusion, the menopausal transition represents a crucial, yet understudied, window of increased cardiovascular risk in women. This prospective study provides evidence that a healthy lifestyle during the midlife, characterized by abstinence from smoking, a healthy diet, and engagement in regular physical activity, is associated with less subclinical atherosclerosis. We further documented that the prevalence of these healthy behaviors is extremely low in midlife women. Thus, this work highlights the growing recognition of the midlife as a critical window for CVD prevention and strongly supports the need for lifestyle interventions aimed at promoting these modifiable health behaviors in midlife women.

## Sources of Funding

The Study of Women's Health Across the Nation has grant support from the NIH through the National Institute on Aging (NIA), the National Institute of Nursing Research (NINR), and the NIH Office of Research on Women's Health (ORWH) (Grants U01NR004061, U01AG012505, U01AG012535, U01AG012531, U01AG012539, U01AG012546, U01AG012553, U01AG012554, and U01AG012495). The content of this article is solely the responsibility of the authors and does not necessarily represent the official views of the NIA, NINR, ORWH, or the NIH.

## Disclosures

None.

## Supporting information


**Table S1.** Alternate Healthy Eating Index (AHEI) Scores in SWAN
**Table S2.** Average HLS and Measures of Subclinical Carotid Atherosclerosis Among 1143 SWAN Participants
**Table S3.** Long‐Term Smoking Status and Measures of Subclinical Carotid Atherosclerosis Among 1143 SWAN Participants
**Table S4.** Average AHEI Score and Measures of Subclinical Carotid Atherosclerosis Among 1143 SWAN Participants
**Table S5.** Long‐Term Physical Activity Status and Measures of Subclinical Carotid Atherosclerosis Among 1143 SWAN Participants
**Table S6.** Quartiles of the Weighted HLS and Measures of Subclinical Carotid Atherosclerosis Among 1143 SWAN Participants
