# Supplementary material for: Healthy Lifestyle During the Midlife Is Prospectively Associated With Less Subclinical Carotid Atherosclerosis: The Study of Women's Health Across the Nation
Source: J Am Heart Assoc. 2018 Nov 28;7(23):e010405. doi: 10.1161/JAHA.118.010405 (PMC6405552; doi:10.1161/JAHA.118.010405)
Supplement: Supplementary file 1 — Table S1. Alternate Healthy Eating Index (AHEI) Scores in SWAN Table S2. Average HLS and Measures of Subclinical Carotid Atherosclerosis Among 1143 SWAN Participants Table S3. Long‐Term Smoking Status and Measures of Subclinical Carotid Atherosclerosis Among 1143 SWAN Participants Table S4. Average AHEI Score and Measures of Subclinical Carotid Atherosclerosis Among 1143 SWAN Participants Table S5. Long‐Term Physical Activity Status and Measures of Subclinical Carotid Atherosclerosis Among 1143 SWAN Participants Table S6. Quartiles of the Weighted HLS and Measures of Subclinical Carotid Atherosclerosis Among 1143 SWAN Participants [file JAH3-7-e010405-s001.pdf]

SUPPLEMENTAL MATERIAL

Table S1. Alternate Healthy Eating Index (AHEI) scores in SWAN.

	Criteria for a minimum score of 0*	Criteria for a maximum score of 10*	AHEI scores† in SWAN (Baseline, 1996-1997)	AHEI scores† in SWAN (Visit 5, 2001-2003)	AHEI scores† in SWAN (Visit 9, 2005-2007)
Vegetables (servings/day)	0	5	3.7 (2.4)	4.1 (2.5)	4.3 (2.5)
Fruit (servings/day)	0	4	3.4 (2.1)	3.2 (2.0)	3.1 (1.9)
Nuts and soy protein (servings/day)	0	1	5.9 (2.7)	6.2 (2.7)	6.4 (2.8)
The ratio of white to red meat	0	4	4.3 (2.9)	4.5 (2.9)	4.7 (2.9)
Cereal fiber (g/day)	0	15	2.9 (1.4)	2.6 (1.3)	2.5 (1.4)
trans fat (% of energy)	≥4	≤0.5	3.7 (3.0)	4.1 (3.0)	4.4 (2.9)
P:S	≤0.1	≥1	6.4 (2.2)	6.6 (2.2)	6.7 (2.3)
Duration of multivitamin use‡	<5y	≥5y	3.4 (1.9)	3.7 (2.2)	4.4 (2.4)
Alcohol (servings/day)	0 or >2.5	0.5-1.5	2.7 (3.7)	2.9 (3.8)	3.1 (3.9)
Total score (range)	2.5	87.5	36.4 (10.1) (11.0-69.6)	37.9 (9.9) (11.2-73.6)	39.5 (10.5) (15.1-74.3)

P:S, the ratio of polyunsaturated to saturated fatty acids; SWAN, Study of Women's Health Across the Nation.

* Intermediate values were scored proportionally between the minimum score and the maximum score.

† Values are mean (standard deviation).

‡ Duration of multivitamin intake was a binary component. The minimum score was 2.5, and the maximum score was 7.5.

Table S2. Average HLS and measures of subclinical carotid atherosclerosis among 1,143 SWAN participants.

	0 to 2		>2 to 3		>3 to 4		>4 to 6		% of SD difference *	*P* Value
n (%)	256 (22.40)		293 (25.63)		306 (26.77)		288 (25.20)			
CCA-IMT, mm	LS-mean	95% CI	LS-mean	95% CI	LS-mean	95% CI	LS-mean	95% CI		
Crude	0.961	0.945, 0.978	0.933§	0.918, 0.949	0.904#	0.889, 0.920	0.898#	0.883, 0.914	-46%	< 0.001
Adjusted 1†	0.958	0.938, 0.979	0.942	0.923, 0.962	0.918#	0.898, 0.938	0.917‖	0.895, 0.940	-30%	< 0.001
Adjusted 2‡	0.954	0.933, 0.975	0.943	0.922, 0.964	0.923‖	0.902, 0.944	0.930	0.907, 0.953	-17%	0.0031
CCA-AD, mm	LS-mean	95% CI	LS-mean	95% CI	LS-mean	95% CI	LS-mean	95% CI		
Crude	7.38	7.30, 7.46	7.19#	7.12, 7.27	7.12#	7.05, 7.19	7.08#	7.00, 7.15	-45%	< 0.001
Adjusted 1†	7.42	7.32, 7.52	7.28‖	7.18, 7.37	7.22#	7.12, 7.32	7.19#	7.08, 7.29	-36%	< 0.001
Adjusted 2‡	7.38	7.28, 7.48	7.26§	7.16, 7.36	7.22‖	7.12, 7.32	7.22‖	7.11, 7.33	-24%	< 0.001
Carotid plaque	OR	95% CI	OR	95% CI	OR	95% CI	OR	95% CI		
Crude	1.00	*Ref*	0.84	0.61, 1.16	0.71§	0.51, 0.97	0.72	0.52, 1.00	-	0.024

Adjusted 1[†]	1.00	*Ref*	0.86	0.61, 1.20	0.67[§]	0.48, 0.95	0.68[§]	0.47, 0.98	-	0.024
Adjusted 2[‡]	1.00	*Ref*	0.88	0.62, 1.24	0.75	0.52, 1.06	0.82	0.56, 1.21	-	0.25

Values are least squares means (95% confidence intervals) for CCA-IMT/CCA-AD from linear models and odds ratios (95% confidence intervals) for carotid plaque index (high vs. moderate vs. none) from cumulative logit models. *P* values were computed by using the HLS as a continuous variable. AD, adventitial diameter; CCA, common carotid artery; CI, confidence interval; HLS, Healthy Lifestyle Score; IMT, intima-media thickness; LS-mean, least squares mean; OR, odds ratio; SD, standard deviation; SWAN, Study of Women's Health Across the Nation.

[*] The difference in the least squares means (by percent of the standard deviation of CCA-IMT/CCA-AD in the study population) comparing the >4 to 6 group to the 0 to 2 group.

[†] Adjusted for baseline covariates, age at the carotid scan (continuous), use of hormone therapy from baseline to the visit of the carotid scan (ever or never), hot flash at Visit 12 (binary), and the number of missing visits for HLS (0, 1, or 2). The baseline covariates include race/ethnicity (African American, Hispanic, Chinese, or non-Hispanic white), education level (≤ high school, some college, or college degree/post-college), financial strain (somewhat/very hard paying for basics, or not hard paying for basics), marital status (single/never married, married/living as if married, or separated/widowed/divorced), self-rated overall health (excellent/very good, good, or fair/poor), Center for Epidemiological Studies Depression scale (≥16 or <16), total energy intake (continuous), menopausal status (premenopausal or early perimenopausal).

‡ Adjusted 1 plus body mass index (continuous), high blood pressure (binary), impaired fasting glucose (binary), serum triglycerides (continuous), total cholesterol (continuous), HDL cholesterol (continuous), LDL cholesterol (continuous), use of antilipidemic medications (binary), and use of antihypertensive medications (binary). The baseline values of these covariates were used except for antilipidemic and antihypertensive medications, for which the values at Visit 12 were used.

§ P<0.05 (compared to the 0 to 2 group).

‖ P<0.01 (compared to the 0 to 2 group).

# P<0.001 (compared to the 0 to 2 group).

Table S3. Long-term smoking status and measures of subclinical carotid atherosclerosis among 1,143 SWAN participants.

	Current smoking at some point		Past smoking		Never smoking		% of SD difference*	*P*-trend
n (%)	158 (13.82)		274 (23.97)		711 (62.20)			
CCA-IMT, mm	LS-mean	95% CI	LS-mean	95% CI	LS-mean	95% CI		
Crude	0.980	0.959, 1.001	0.906§	0.890, 0.923	0.917§	0.907, 0.927	-46%	< 0.001
Adjusted†	0.976	0.952, 1.001	0.922§	0.899, 0.945	0.929§	0.912, 0.947	-34%	0.0026
CCA-AD, mm	LS-mean	95% CI	LS-mean	95% CI	LS-mean	95% CI		
Crude	7.40	7.30, 7.50	7.15§	7.08, 7.23	7.15§	7.10, 7.20	-38%	< 0.001
Adjusted†	7.46	7.35, 7.58	7.26‡	7.15, 7.37	7.22§	7.14, 7.31	-36%	< 0.001
Carotid plaque	OR	95% CI	OR	95% CI	OR	95% CI		
Crude	1.00	*Ref*	0.55‡	0.38, 0.80	0.55§	0.40, 0.76	-	0.0028
Adjusted†	1.00	*Ref*	0.45§	0.30, 0.69	0.51§	0.35, 0.73	-	0.0064

Long-term smoking status was summarized using the available visits from baseline, Visit 5, and Visit 9. Values are least squares means (95% confidence intervals) for CCA-IMT/CCA-AD from linear models and odds ratios (95% confidence intervals) for carotid plaque index (high vs. moderate vs. none) from cumulative logit models. *P*-trend values were computed by assigning 0, 1, and 2 to the three groups, respectively, while treating it as a continuous variable. AD, adventitial diameter;

CCA, common carotid artery; CI, confidence interval; IMT, intima-media thickness; LS-mean, least squares mean; OR, odds ratio; SD, standard deviation; SWAN, Study of Women's Health Across the Nation.

[*] The difference in the least squares means (by percent of the standard deviation of CCA-IMT/CCA-AD in the study population) comparing the "never smoking" group to the "current smoking at some point" group.

[†] Adjusted for baseline covariates, age at the carotid scan (continuous), use of hormone therapy from baseline to the visit of the carotid scan (ever or never), hot flash at Visit 12 (binary), the number of missing visits for HLS (0, 1, or 2), long-term physical activity (consistently not meeting the recommendation, mixture of not meeting and partially meeting the recommendation, or fully meeting the recommendation at some point), and average alternate healthy eating index score (tertiles). The baseline covariates include race/ethnicity (African American, Hispanic, Chinese, or non-Hispanic white), education level (≤ high school, some college, or college degree/post-college), financial strain (somewhat/very hard paying for basics, or not hard paying for basics), marital status (single/never married, married/living as if married, or separated/widowed/divorced), self-rated overall health (excellent/very good, good, or fair/poor), Center for Epidemiological Studies Depression scale (≥16 or <16), total energy intake (continuous), menopausal status (premenopausal or early perimenopausal), body mass index (continuous), high blood pressure (binary), impaired fasting glucose (binary), serum triglycerides (continuous), total cholesterol (continuous), HDL cholesterol (continuous), LDL cholesterol (continuous), use of antilipidemic medications (binary), and use of antihypertensive medications (binary). The baseline values of these covariates were used except for antilipidemic and antihypertensive medications, for which the values at Visit 12 were used.

[‡] $P<0.01$ (compared to the "current at some point" group).

[§] $P<0.001$ (compared to the "current at some point" group).

Table S4. Average AHEI score and measures of subclinical carotid atherosclerosis among 1,143 SWAN participants.

	Tertile 1		Tertile 2		Tertile 3		% of SD difference[*]	*P*-trend
n (%)	381 (33.33)		381 (33.33)		381 (33.33)			
CCA-IMT, mm	LS-mean	95% CI	LS-mean	95% CI	LS-mean	95% CI		
Crude	0.948	0.934, 0.962	0.919[#]	0.905, 0.932	0.903[**]	0.889, 0.916	-33%	< 0.001
Adjusted 1[†]	0.952	0.932, 0.972	0.934	0.914, 0.953	0.928[‖]	0.908, 0.948	-18%	0.019
Adjusted 2[‡]	0.956	0.935, 0.977	0.941	0.921, 0.962	0.937	0.916, 0.958	-14%	0.067
Adjusted 3[§]	0.952	0.931, 0.973	0.938	0.918, 0.959	0.938	0.917, 0.959	-10%	0.18
CCA-AD, mm	LS-mean	95% CI	LS-mean	95% CI	LS-mean	95% CI		
Crude	7.30	7.24, 7.37	7.15[#]	7.08, 7.21	7.10[**]	7.04, 7.17	-30%	< 0.001
Adjusted 1[†]	7.40	7.30, 7.49	7.29[‖]	7.20, 7.39	7.26[#]	7.16, 7.36	-21%	0.0072
Adjusted 2[‡]	7.40	7.30, 7.51	7.31[‖]	7.21, 7.41	7.28[‖]	7.18, 7.39	-18%	0.016
Adjusted 3[§]	7.37	7.27, 7.47	7.29	7.19, 7.38	7.29	7.19, 7.39	-12%	0.11
Carotid plaque	OR	95% CI	OR	95% CI	OR	95% CI		

Crude	1.00	*Ref*	0.87	0.66, 1.14	0.89	0.67, 1.17	-	0.40
Adjusted 1[†]	1.00	*Ref*	0.92	0.69, 1.23	0.91	0.67, 1.23	-	0.53
Adjusted 2[‡]	1.00	*Ref*	1.00	0.74, 1.35	1.00	0.73, 1.37	-	0.99
Adjusted 3[§]	1.00	*Ref*	1.01	0.75, 1.35	1.02	0.74, 1.40	-	0.91

Average AHEI score was calculated using the available visits from baseline, Visit 5, and Visit 9. Values are least squares means (95% confidence intervals) for CCA-IMT/CCA-AD from linear models and odds ratios (95% confidence intervals) for carotid plaque index (high vs. moderate vs. none) from cumulative logit models. *P*-trend values were computed by assigning the median AHEI of a level to participants in the corresponding level and treating it as a continuous variable. AD, adventitial diameter; AHEI, Alternate Healthy Eating Index; CCA, common carotid artery; CI, confidence interval; IMT, intima-media thickness; LS-mean, least squares mean; OR, odds ratio; SD, standard deviation; SWAN, Study of Women's Health Across the Nation.

[*] The difference in the least squares means (by percent of the standard deviation of CCA-IMT/CCA-AD in the study population) comparing the third tertile to the first tertile.

[†] Adjusted for baseline covariates, age at the carotid scan (continuous), use of hormone therapy from baseline to the visit of the carotid scan (ever or never), hot flash at Visit 12 (binary), the number of missing visits for HLS (0, 1, or 2), long-term smoking status (current smoking at some point, past smoking, or never smoking), and levels of long-term physical activity (consistently not meeting the recommendation, mixture of not meeting and partially meeting the recommendation, or fully meeting the recommendation at some point). The baseline covariates include race/ethnicity (African American, Hispanic, Chinese, or non-

Hispanic white), education level (≤ high school, some college, or college degree/post-college), financial strain (somewhat/very hard paying for basics, or not hard paying for basics), marital status (single/never married, married/living as if married, or separated/widowed/divorced), self-rated overall health (excellent/very good, good, or fair/poor), Center for Epidemiological Studies Depression scale (≥16 or <16), total energy intake (continuous), and menopausal status (premenopausal or early perimenopausal).

‡ Adjusted 1 plus high blood pressure (binary), impaired fasting glucose (binary), serum triglycerides (continuous), total cholesterol (continuous), HDL cholesterol (continuous), LDL cholesterol (continuous), use of antilipidemic medications (binary), and use of antihypertensive medications (binary). The baseline values of these covariates were used except for antilipidemic and antihypertensive medications, for which the values at Visit 12 were used.

§ Adjusted 2 plus baseline body mass index (continuous).

‖ $P<0.05$ (compared to the first tertile).

# $P<0.01$ (compared to the first tertile).

** $P<0.001$ (compared to the first tertile).

Table S5. Long-term physical activity status and measures of subclinical carotid atherosclerosis among 1,143 SWAN participants.

	Consistently not meeting the recommendation		Mixture of not meeting and partially meeting the recommendation		Fully meeting the recommendation at some point		% of SD difference *	*P*-trend
n (%)	268 (23.45)		517 (45.23)		358 (31.32)			
CCA-IMT, mm	LS-mean	95% CI	LS-mean	95% CI	LS-mean	95% CI		
Crude	0.951	0.934, 0.967	0.926[‖]	0.914, 0.938	0.898[#]	0.884, 0.912	-38%	< 0.001
Adjusted 1[†]	0.950	0.931, 0.970	0.937	0.918, 0.955	0.927	0.905, 0.950	-17%	0.059
Adjusted 2[‡]	0.953	0.933, 0.974	0.941	0.922, 0.961	0.939	0.916, 0.963	-10%	0.26
Adjusted 3[§]	0.949	0.928, 0.970	0.940	0.920, 0.959	0.939	0.916, 0.962	-7%	0.43
CCA-AD, mm	LS-mean	95% CI	LS-mean	95% CI	LS-mean	95% CI		
Crude	7.27	7.19, 7.35	7.22	7.16, 7.27	7.08[#]	7.01, 7.15	-28%	< 0.001
Adjusted 1[†]	7.34	7.24, 7.44	7.33	7.23, 7.42	7.28	7.17, 7.39	-9%	0.31
Adjusted 2[‡]	7.35	7.25, 7.45	7.33	7.24, 7.43	7.32	7.21, 7.43	-4%	0.64

Adjusted 3[§]	7.31	7.21, 7.41	7.32	7.22, 7.41	7.32	7.21, 7.43	+1%	0.91
Carotid plaque	OR	95% CI	OR	95% CI	OR	95% CI		
Crude	1.00	*Ref*	0.77	0.58, 1.02	0.88	0.65, 1.19	-	0.53
Adjusted 1[†]	1.00	*Ref*	0.73[‖]	0.53, 0.99	0.83	0.58, 1.19	-	0.47
Adjusted 2[‡]	1.00	*Ref*	0.72[‖]	0.52, 0.99	0.94	0.65, 1.36	-	0.98
Adjusted 3[§]	1.00	*Ref*	0.72[‖]	0.53, 1.00	0.96	0.66, 1.38	-	0.92

Long-term physical activity status was summarized using the available visits from baseline, Visit 5, and Visit 9. Values are least squares means (95% confidence intervals) for CCA-IMT/CCA-AD from linear models and odds ratios (95% confidence intervals) for carotid plaque index (high vs. moderate vs. none) from cumulative logit models. *P*-trend values were computed by assigning 0, 1, and 2 to the three groups, respectively, while treating it as a continuous variable. AD, adventitial diameter; CCA, common carotid artery; CI, confidence interval; IMT, intima-media thickness; LS-mean, least squares mean; OR, odds ratio; SD, standard deviation; SWAN, Study of Women's Health Across the Nation.

[*] The difference in the least squares means (by percent of the standard deviation of CCA-IMT/CCA-AD in the study population) comparing the "fully meeting the recommendation at some point" group to the "consistently not meeting the recommendation" group.

[†] Adjusted for baseline covariates, age at the carotid scan (continuous), use of hormone therapy from baseline to the visit of the carotid scan (ever or never), hot flash at Visit 12 (binary), the number of missing visits for HLS (0, 1, or 2), long-term smoking

status (current smoking at some point, past smoking, or never smoking), and average alternate healthy eating index (tertiles). The baseline covariates include race/ethnicity (African American, Hispanic, Chinese, or non-Hispanic white), education level (≤ high school, some college, or college degree/post-college), financial strain (somewhat/very hard paying for basics, or not hard paying for basics), marital status (single/never married, married/living as if married, or separated/widowed/divorced), self-rated overall health (excellent/very good, good, or fair/poor), Center for Epidemiological Studies Depression scale (≥16 or <16), total energy intake (continuous), and menopausal status (premenopausal or early perimenopausal).

[‡] Adjusted 1 plus high blood pressure (binary), impaired fasting glucose (binary), serum triglycerides (continuous), total cholesterol (continuous), HDL cholesterol (continuous), LDL cholesterol (continuous), use of antilipidemic medications (binary), and use of antihypertensive medications (binary). The baseline values of these covariates were used except for antilipidemic and antihypertensive medications, for which the values at Visit 12 were used.

[§] Adjusted 2 plus baseline body mass index (continuous).

[‖] $P<0.05$ (compared to the “consistently not meeting the recommendation” group).

[#] $P<0.001$ (compared to the “consistently not meeting the recommendation” group).

Table S6. Quartiles of the weighted HLS and measures of subclinical carotid atherosclerosis among 1,143 SWAN participants.

	Quartile 1		Quartile 2		Quartile 3		Quartile 4		% of SD difference *	*P* Value
CCA-IMT, mm	LS-mean	95% CI	LS-mean	95% CI	LS-mean	95% CI	LS-mean	95% CI		
Crude	0.960	0.944, 0.976	0.930[‖]	0.914, 0.946	0.904[#]	0.888, 0.919	0.898[#]	0.882, 0.914	-45%	< 0.001
Adjusted 1[†]	0.953	0.938, 0.977	0.936	0.916, 0.955	0.918[#]	0.897, 0.939	0.917[‖]	0.894, 0.940	-29%	< 0.001
Adjusted 2[‡]	0.953	0.933, 0.974	0.938	0.918, 0.959	0.923[‖]	0.901, 0.945	0.930	0.906, 0.953	-17%	0.0035
CCA-AD, mm	LS-mean	95% CI	LS-mean	95% CI	LS-mean	95% CI	LS-mean	95% CI		
Crude	7.37	7.29, 7.45	7.20[‖]	7.12, 7.27	7.11[#]	7.03, 7.18	7.07[#]	6.99, 7.14	-46%	< 0.001
Adjusted 1[†]	7.42	7.33, 7.52	7.29[§]	7.19, 7.39	7.19[#]	7.10, 7.29	7.19[#]	7.09, 7.30	-35%	< 0.001
Adjusted 2[‡]	7.38	7.28, 7.48	7.28[§]	7.18, 7.38	7.19[#]	7.09, 7.29	7.23[‖]	7.12, 7.34	-23%	< 0.001
Carotid plaque	OR	95% CI	OR	95% CI	OR	95% CI	OR	95% CI		
Crude	1.00	*Ref*	0.75	0.55, 1.03	0.70[§]	0.51, 0.96	0.67[§]	0.49, 0.92	-	< 0.001
Adjusted 1[†]	1.00	*Ref*	0.75	0.54, 1.05	0.70[§]	0.50, 0.97	0.67[§]	0.48, 0.94	-	< 0.001
Adjusted 2[‡]	1.00	*Ref*	0.75	0.54, 1.06	0.68[§]	0.48, 0.96	0.68[§]	0.48, 0.96	-	< 0.001

The weighted HLS was generated by using the percentage of the coefficient of each individual component to the sum of the coefficients in the models with all components included. Physical activity was included both as a linear and a quadratic term in the creation of the weighed HLS. Values are least squares means (95% confidence intervals) for CCA-IMT/CCA-AD from linear models and odds ratios (95% confidence intervals) for carotid plaque index (high vs. moderate vs. none) from cumulative logit models. *P* values were computed by using the HLS as a continuous variable. AD, adventitial diameter; CCA, common carotid artery; CI, confidence interval; HLS, Healthy Lifestyle Score; IMT, intima-media thickness; LS-mean, least squares mean; OR, odds ratio; SD, standard deviation; SWAN, Study of Women's Health Across the Nation.

[*] The difference in the least squares means (by percent of the standard deviation of CCA-IMT/CCA-AD in the study population) comparing highest quartile to the lowest quartile.

[†] Adjusted for baseline covariates, age at the carotid scan (continuous), use of hormone therapy from baseline to the visit of the carotid scan (ever or never), hot flash at Visit 12 (binary), and the number of missing visits for HLS (0, 1, or 2). The baseline covariates include race/ethnicity (African American, Hispanic, Chinese, or non-Hispanic white), education level (≤ high school, some college, or college degree/post-college), financial strain (somewhat/very hard paying for basics, or not hard paying for basics), marital status (single/never married, married/living as if married, or separated/widowed/divorced), self-rated overall health (excellent/very good, good, or fair/poor), Center for Epidemiological Studies Depression scale (≥16 or <16), total energy intake (continuous), menopausal status (premenopausal or early perimenopausal).

‡ Adjusted 1 plus body mass index (continuous), high blood pressure (binary), impaired fasting glucose (binary), serum triglycerides (continuous), total cholesterol (continuous), HDL cholesterol (continuous), LDL cholesterol (continuous), use of antilipidemic medications (binary), and use of antihypertensive medications (binary). The baseline values of these covariates were used except for antilipidemic and antihypertensive medications, for which the values at Visit 12 were used.

§ $P<0.05$ (compared to the first quartile).

|| $P<0.01$ (compared to the first quartile).

# $P<0.001$ (compared to the first quartile).
